# The effects of trastuzumab on the CD4+CD25+FoxP3+ and CD4+IL17A+ T-cell axis in patients with breast cancer

**DOI:** 10.1038/sj.bjc.6604963

**Published:** 2009-03-10

**Authors:** C Horlock, B Stott, P J Dyson, M Morishita, R C Coombes, P Savage, J Stebbing

**Affiliations:** 1 Department of Oncology, Imperial College School of Medicine, Hammersmith Hospital Campus, Du Cane Road, London W12 ONN, UK; 2 Department of Immunology, Imperial College School of Medicine, Hammersmith Hospital Campus, Du Cane Road, London W12 ONN, UK; 3 Department of Medical Oncology, Imperial College Healthcare NHS Trust, Charing Cross Hospital, Fulham Palace Road, London, W6 8RF, UK

**Keywords:** trastuzumab, T_reg_, Th17, HER2, immunology

## Abstract

In addition to the direct targeting effects on HER2-positive cells, trastuzumab may have a therapeutic role modulating the activity of the cellular immune system in patients with breast cancer. To investigate this further, the balance of T-regulatory (T_reg_), Th17, natural killer (NK) and NK T (NKT) cells before, during and after trastuzumab therapy was investigated. Sequential frequencies of circulating T_reg_ cells, Th17 cells, NK and NKT cells were measured in peripheral blood of breast cancer patients and normal controls throughout therapy. Individuals with breast cancer had significantly higher T_reg_ frequencies of peripheral blood compared with healthy controls (9.2 or 8.6 *vs* 6%; *P*<0.05), and no significant differences in T_reg_ frequencies were observed between HER2-positive and HER2-negative individuals. The number of Th17 cells was lowest in HER2-positive patients compared with both healthy controls and HER2-negative patients (0.31 *vs* 0.75% or 0.84%; *P*=0.01). There appeared to be an inverse relationship between T_reg_ and Th17 frequencies in metastatic breast cancer (MBC) with T_reg_ levels significantly reduced during treatment with trastuzumab (*P*=0.04), whereas Th17 frequencies were concomitantly increased (*P*=0.04). This study supports earlier data that T_reg_ cells are present at higher frequencies in breast cancer patients compared with healthy individuals. For the first time, we show that HER2-positive individuals with breast carcinomas have reduced numbers of circulating Th17 cells, which appear, in turn to have an inverse relationship with T_reg_ frequency in MBC. The change in balance of the T_reg_ : Th17 ratio appears to characterise the cancer state, and furthermore, is disrupted by trastuzumab therapy.

Several studies have shown that higher numbers of T-regulatory (T_reg_) cells are associated with progression in a variety of malignancies, and T_reg_ numbers appear increased in individuals with a variety of solid tumours (lung, breast and pancreas) and haematological malignancies, and this is reflected in the peripheral blood (PB) of cancer patients (Woo *et al*, 2001; Liyanage *et al*, 2002; Diederichsen *et al*, 2003; Javia and Rosenberg, 2003; Wolf *et al*, 2003; Marshall *et al*, 2004; Galon *et al*, 2006; Nedergaard *et al*, 2007). The T_reg_ subgroup of immune cells suppresses the activity of effector cells, including CD4+ and CD8+ cytotoxic T cells, natural killer (NK) cells, dendritic cells, NK T (NKT) cells and B cells (Murakami *et al*, 2002; Trzonkowski *et al*, 2004). The outcome of this activity appears to promote the survival of cancer cells by affording protection from both the innate and adaptive immune systems.

Th17 cells have been defined as an immune subset of CD4+ lymphocytes, characterised by their production of the cytokines IL6, IL17 and TNF-*α* and expression of the transcription factor, RORγT. They have been implicated in inflammation and autoimmune disease, but little is known about their prevalence and function in human cancer. Initial reports have identified Th17s within ovarian tumour (Kryczek *et al*, 2007; Miyahara *et al*, 2008), but their precise role in this setting remains unclear.

The differentiation of Th17 cells is promoted by IL-23, an inducer of experimental autoimmune encephalitis (Langrish *et al*, 2005; McKenzie *et al*, 2006), and it is thought that TGF-*β* plays a key role in determining whether CD4+ lymphocytes become T_reg_ or Th17 cells *in vitro* and possibly *in vivo* (Veldhoen *et al*, 2006; Zhou *et al*, 2008). The balance of T_reg_ cells and Th17s may influence the immune response against cancer, with a T_reg_ bias favouring tumour immune escape.

There are increasing data that the activity of regulatory cells controlling the cellular immune system may have importance in the clinical outcomes of cancer therapies (Bamias *et al*, 2008). Some chemotherapeutic agents, such as cyclophosphamide, have been shown to lead to reduced numbers of functional T_reg_ cells (Beyer and Schultze, 2006). Conversely, those treated with high-dose interleukin-2 (IL-2) for melanoma and renal cell carcinoma show expansion of these cells (Ahmadzadeh and Rosenberg, 2006).

In breast cancer, it is unclear whether trastuzumab, an IgG_1_ monoclonal antibody, has direct effects on T_reg_ immune subsets. Several studies have shown an increased prevalence of circulating T_reg_ cells in breast cancer patients compared with normal controls (Liyanage *et al*, 2002; Perez *et al*, 2007), and the presence of T_reg_ cells is likely to correlate to cancer progression. However, the effect of breast cancer therapies, such as trastuzumab, upon T_reg_ cells and other immune subsets remains unknown. Here, we have examined the numbers of T_reg_ cells, Th17, NK and NKT cells in Her-2/neu (HER2)-positive (early and metastatic breast cancer (MBC)) and HER2-negative breast cancer patients, with the aim of further elucidation of the role of T_reg_ and Th17 cells, and their balance, in different patient populations with breast cancer over time.

## PATIENTS AND METHODS

### Patients

Between September 2007 and January 2009, patients with HER2-positive breast cancer were recruited before therapy with trastuzumab. Individuals with negative HER2 and healthy controls were also recruited. The study obtained appropriate ethical approval in accordance with the Declaration of Helsinki. Peripheral blood was collected before and during therapy into sodium heparin tubes, and PB mononuclear cells (PBMCs) were isolated by density centrifugation, and frozen in liquid nitrogen until analysis.

Patients receiving adjuvant trastuzumab had completed cytotoxic chemotherapy ⩾3 weeks before the baseline blood sample, and then went on to receive trastuzumab monotherapy. The majority of individuals whom we studied receiving trastuzumab for metastatic disease also received monotherapy, but a minority were treated in combination with docetaxel or vinorelbine. Samples were taken before the beginning of the treatment, during the treatment (one or more time points), and for early breast cancer (EBC) patients, samples were also taken following the completion of the course of adjuvant trastuzumab. In addition, samples from seven HER2-negative patients and four healthy donors (all age-matched females) were also obtained. As we treat patients with metastatic cancer with trastuzumab beyond progression, we did not obtain ‘metastatic samples’ post-treatment for this study.

### Cell staining

Before intracellular staining, PBMCs were thawed and restimulated for 4 h with 50 ng ml^−1^ phorbol 12-myristate 13-acetate (PMA) and 100 ng ml^−1^ ionomycin in the presence of (10 *μ*g ml^−1^) protein transport inhibitor Brefeldin A (Sigma, Poole, UK). Cells were surface stained with combinations of CD4-APC (clone RPA-T4), CD3-PE (UCHT1), CD25-FITC (BC96), CD45RO-FITC (UCHL1), CD56-FITC (MEM188), GITR-PE (ebioAITR) and CTLA-4-PE (14D3; all antibodies from eBioscience, San Diego, CA, USA). Cells were fixed and permeabilised with the FoxP3 fix/perm kit (eBioscience), and intracellularly stained with PE-conjugated antibodies against FoxP3 and IL-17A (clones PCH101 and eBio64DEC17, respectively, from eBioscience). Isotype control antibodies were used to determine the background fluorescence.

### Flow cytometry analysis

Peripheral blood mononuclear cells were analysed on a FACScalibur flow cytometer equipped with CellQuest software (BD Biosciences, San Jose, CA, USA), and data were analysed using FlowJo v. 7 (Treestar Inc., Ashland, OR, USA). Regulatory T cells were defined as CD4+FoxP3+CD25+, and the expression of GITR and CTLA were also measured. Th17 cells were defined as CD4+IL17A+; NK cells were defined as CD3-CD56+, whereas NKT cells were counted as CD3+CD56+ cells.

### Statistics

All statistical comparisons between sample groups were carried out using the non-parametric Mann–Whitney *U*-test with *P*-values <0.05 considered significant. For patients who died, we used the last observation carried forward for statistical analyses.

## RESULTS

### Breast cancer patients have increased numbers of T_reg_ cells

A total of 27 patients with HER2-positive breast cancer were recruited. All of these individuals were being treated with trastuzumab, either in the adjuvant setting for EBC (*n*=14; mean age 53 years, range: 33–64) or for MBC (*n*=13; mean age 58, range 32–79). During the course of this study, two HER2-positive patients died.

**Figure 1:**
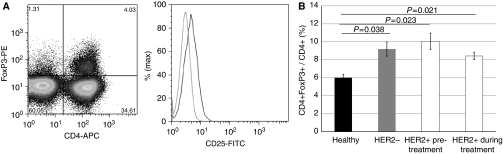
T_reg_ frequency is increased in breast cancer patients compared with healthy individuals. (**A**) A representative T_reg_ FACS plot showing CD4+FoxP3+ cells. By gating on the CD4+FoxP3+ cells, it was shown that they were also CD25 expressing (shown in histogram; key to histogram: isotype control (dotted grey line), CD4+FoxP3- cells (grey line), CD4+FoxP3+ cells (black line)). (**B**) Comparison of T_reg_ frequencies in healthy individuals (black), HER2− breast cancer patients (grey) and HER2+ breast cancer patients, either before or during treatment with trastuzumab (white). Shown is the frequency of T_reg_ cells as a percentage of total CD4+ cells. Error bars represent±s.e. *P*-values < 0.05, as measured by the Mann–Whitney *U*-test, were considered significant.

Initially, a comparison of cell frequencies between HER2-positive and HER2-negative breast cancer patients was undertaken. Regulatory T cells were characterised as CD4+FoxP3+; in addition, the expression of CD25, GITR and CTLA-4 among this population was also confirmed (Figure 1A). The absolute numbers and percentage of T_reg_ cells in the PB of healthy individuals and breast cancer patients was determined, and our data show that individuals with breast cancer have a significantly higher T_reg_ frequency in the PB compared with healthy controls; T_reg_ frequencies were >8% of CD4+ cells in breast cancer, compared with 6% of CD4+ cells in healthy controls (*P*<0.05). The frequency of T_reg_ cells precisely reflected the absolute numbers of T_reg_ cells observed (as measured as the number of T_reg_ cells per million PBMCs counted; Supplementary data figure).

To further investigate the role of HER2 status and therapy, we divided patients into HER2-positive or HER2-negative groups. Our data showed that patients with HER2-positive breast cancer had significantly higher frequencies of T_reg_ cells compared with healthy individuals (8.4 *vs* 6%; *P*=0.021), whereas the frequency was similar for both HER2-positive and HER2-negative patients (8.4 *vs* 9.2%; *P*=0.27; Figure 1B). HER2-positive patients during treatment with trastuzumab had a reduced frequency of T_reg_ cells, compared with that seen at baseline before therapy, but this difference was not statistically significant.

### Numbers of Th17 cells are decreased in HER2-positive breast cancer patients

**Figure 2:**
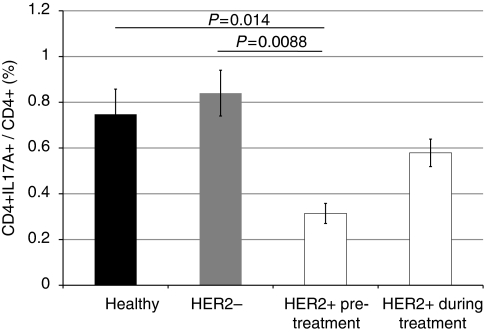
The number of circulatory Th17 cells is decreased in HER2+ breast cancer patients. The frequency of Th17 cells in HER2− breast cancer patients (grey) did not differ from that measured in healthy individuals (black). HER2+ breast cancer patients (white) had significantly lower Th17 frequency compared with both healthy individuals and HER2− patients, and the frequency of Th17s increased during trastuzumab therapy. Th17 frequency was measured as the frequency of CD4+IL17A+ T cells as a percentage of total CD4+ cells. Error bars represent±s.e. *P*-values < 0.05, as measured by the Mann–Whitney *U*-test, were considered significant.

As for the T_reg_ cells, the frequency and absolute number of Th17 cells were measured and compared in healthy individuals and HER2-positive and -negative breast cancer patients (Figure 2 and Supplementary data). Healthy individuals and HER2-negative breast cancer patients had similar Th17 numbers, and in an inverse relationship than that observed in T_reg_ cells, HER2-positive patients had significantly lower frequencies of Th17s compared with healthy and HER2-negative individuals (0.314% compared with 0.748% (*P*=0.014) and 0.84% (*P*=0.0088), respectively). We observed that the number of Th17 cells increased upon treatment with trastuzumab (0.314% compared with 0.579%; not significant).

### The balance of T_reg_ and Th17 cells in PB is altered in HER2-positive breast cancer

**Figure 3:**
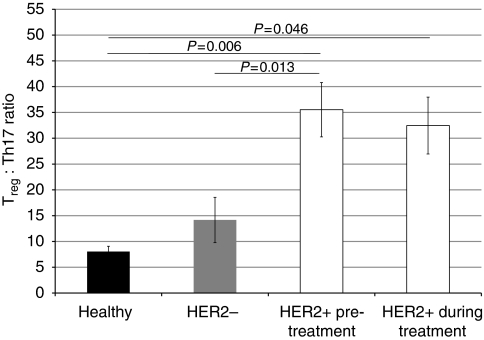
The T_reg_/Th17 bias is highest in HER2+ breast cancer. The T_reg_ : Th17 cell ratio in healthy individuals (black), HER2− breast cancer patients (grey) and HER2+ breast cancer patients treated with trastuzumab (white). The number of T_reg_ cells to Th17s is significantly higher in HER2+ breast cancer patients compared with healthy individuals and HER2− breast cancer patients, with a slight decrease during trastuzumab treatment. The number of T_reg_ cells to every Th17 cell is shown. *P*-values <0.05, as measured by the Mann–Whitney *U*-test, were considered significant.

To further quantify the relationship between T_reg_ and Th17 numbers in patients with breast cancer, the number of T_reg_ cells to every Th17 cell was calculated as a ratio. Here, we found that HER2-positive breast cancer patients had the highest ratio of T_reg_ : Th17 cells (Figure 3; 35.5 : 1, T_reg_ : Th17 compared with 8 : 1 in healthy donors; *P*=0.006); HER2-negative patients had a similar balance of T_reg_ : Th17 compared with healthy donors (14 : 1 compared with 8 : 1; *P*=0.26). Upon treatment with trastuzumab, the T_reg_ : Th17 bias seen in HER2-positive individuals was reduced slightly to 32:1.

### A converse relationship between T_reg_ and Th17 frequency is seen in MBC

**Figure 4:**
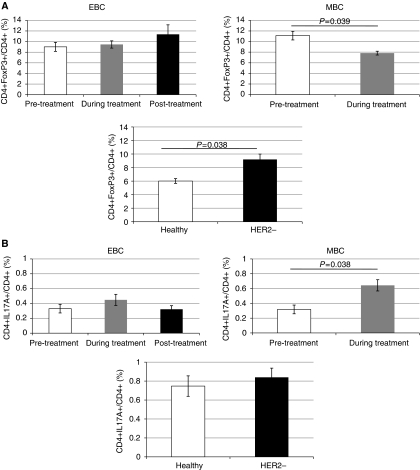
The effect of breast cancer stage and trastuzumab on T_reg_ and Th17 frequencies. The frequency of T_reg_ cells and Th17s was measured pre- (white), during (grey) and post (black)-trastuzumab therapy in early breast cancer (EBC) and metastatic breast cancer (MBC) patients. The third histogram shows the relative frequencies seen in healthy and HER2− controls. (**A**) T_reg_ cells (**B**) Th17s. Trastuzumab therapy in MBC leads to a statistically significant decrease in the frequency of T_reg_ cells, and conversely, a statistically significant increase in Th17s. Error bars represent±s.e. *P*-values < 0.05, as measured by the Mann–Whitney *U*-test, were considered significant.

We then sought to address whether the increase of T_reg_ cells observed in individuals treated with trastuzumab was a ‘drug-specific’ change dependent on cancer stage. Patients were divided into those who had EBC with no metastatic sites, and patients who had at least one metastatic site (MBC). This revealed individuals with EBC had similar T_reg_ frequencies before, and during trastuzumab therapy (9 and 9.5%, respectively; Figure 4A), with a slight increase following the completion of the course of treatment. The T_reg_ frequency in EBC was comparable to that seen in HER2-negative cancer patients (9 *vs* 9.2%; *P*=0.79). Metastatic breast cancer patients had the highest frequency of T_reg_ cells from all populations that we studied (11.1% pre-treatment); this significantly decreased when patients were treated with trastuzumab (7.8%; *P*=0.039).

As for T_reg_ cells, EBC patients had similar Th17 frequencies pre-, during and post-trastuzumab therapy (0.33, 0.45 and 0.32%, respectively). However, in MBC patients, the frequency of Th17s in PB followed an inverse relationship to T_reg_ cell frequency, with numbers significantly increasing during treatment with trastuzumab (0.32 *vs* 0.64%; *P*=0.038). The frequency of Th17 cells in HER2-negative breast cancer patients was comparable with that seen in healthy individuals (0.84 *vs* 0.75%).

**Figure 5:**
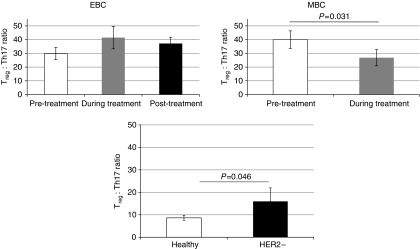
The T_reg_/Th17 balance. The ratio of T_reg_ : Th17 cells was measured pre- (white), during (grey), and post (black)-trastuzumab therapy in early breast cancer (EBC) and metastatic breast cancer (MBC) patients. The third histogram shows the relative ratios seen in healthy and HER2− controls. The number of T_reg_ cells to every Th17 cell is shown. Error bars represent ±s.e. *P*-values <0.05, as measured by the Mann–Whitney U-test, were considered significant.

When quantifying the relationship between T_reg_ and Th17 frequency, we found that of all populations studied, healthy individuals had the lowest number of T_reg_ cells to every Th17 cell (8.6 : 1). This was significantly lower than that seen in HER2-negative breast cancer (16 : 1; *P*=0.046), HER2-positive EBC patients (30 : 1; *P*=0.017) and also HER2-positive MBC patients (40 : 1; *P*=0.014). In EBC, the T_reg_ : Th17 frequency increased during treatment with trastuzumab, then decreased slightly following completion, but not quite to the levels seen before treatment (pre-treatment: 30 : 1, during: 41.4 : 1, post-treatment: 37.1 : 1). In MBC, however, the T_reg_ : Th17 ratio decreased during treatment (40 : 1 *vs* 26.8 : 1; *P*=0.031; Figure 5).

As samples were obtained at multiple time points over the course of trastuzumab treatment, the frequency of cells over time was also measured. The majority of patients remained on the same treatment regime throughout the period of this study, and the frequency of cells (all subsets) remained consistent throughout the specific treatment time course and changed only on starting or stopping the trastuzumab.

### Natural killer and NKT cells in breast cancer

In addition to T_reg_ and Th17 cells, the absolute numbers of circulating NK and NKT cells were also measured. This revealed no significant difference between patient populations. The balances and frequencies of NK : NKT cells and T_reg _: NKT cells were also investigated (as others have performed in inflammatory settings), but again no significant differences were observed.

## DISCUSSION

This study is one of the first to document circulating Th17 cells in human cancer patients. Th17 cells have been found in the human gut and PB, and are thought to play a role in inflammatory and autoimmune disorders (Stockinger and Veldhoen, 2007); but their role in cancer, especially *in vivo*, is however unknown.

We have shown that HER2-positive breast cancer patients have significantly lower frequencies of Th17 cells in their PB compared with both healthy individuals and HER2-negative breast cancer patients. This contradicts a recent study that suggested that although the tumour environment in ovarian cancer was favourable for generation of Th17 cells, the frequencies of Th17 cells in the PB of cancer patients was comparable to that seen in healthy individuals (Miyahara *et al*, 2008). In addition, we showed that in breast cancer, circulating T_reg_ cells are increased compared with healthy controls (data supported by earlier study (Wolf *et al*, 2003)). We also observed that although HER2-positive breast cancer patients have a significantly higher frequency of T_reg_ cells compared with healthy donors; the frequency of T_reg_ cells in HER2-positive and -negative individuals did not differ. This finding is in contrast with the data of Perez *et al* (2007) who found that HER2-negative patients had similar frequencies of T_reg_ cells as healthy donors, but that HER2-positive individuals had significantly higher proportions than both populations.

It is clear that the development of T_reg_ and Th17 cells is closely linked, and both require TGF-*β* for their differentiation from naïve T cells (Ivanov *et al*, 2006; Davidson *et al*, 2007). Recently, the balance of T_reg_ and Th17 cells, and the regulatory balance between these cell types has been of interest to several groups (Elias *et al*, 2008; Quintana *et al*, 2008; Veldhoen *et al*, 2008; Zhou *et al*, 2008). Although both cell types require TGF-*β*, other compounds, such as IL-6 and retinoic acid (Nishihara *et al*, 2007; Wan *et al*, 2007; Elias *et al*, 2008), specifically promote either T_reg_ or Th17 cells while suppressing the other cell type.

To further quantify this relationship, we examined the balance between T_reg_ and Th17 cells, by calculating the ratio of T_reg_ : Th17 cells. It was shown that HER2-positive patients had a much higher T_reg_ : Th17 ratio than both HER2-negative individuals and healthy controls. The T_reg_ : Th17 ratio was significantly reduced in MBC patients during trastuzumab therapy, but no such relationship was observed in EBC patients treated with adjuvant trastuzumab. In addition to the T_reg_ : Th17 axis, a regulatory relationship between T_reg_ and NKT cells has also been described (La Cava *et al*, 2006); the relationship between these cells in the context of breast cancer was therefore examined. We showed no significant differences in NKT cell numbers between all populations, and no significant difference in the T_reg_ : NKT ratio. This suggests that the T_reg_ : Th17 balance ratio is of greater importance than that of the T_reg_ : NKT in breast cancer.

Increasing evidence suggests that the activity of regulatory cells controlling the cellular immune system may have importance in the clinical outcomes of cancer therapies (Bamias *et al*, 2008). Several studies have shown that higher numbers of T_reg_ cells are associated with progression in a variety of malignancies and can correlate to the poor prognosis (Liyanage *et al*, 2002; Galon *et al*, 2006). Here, we suggest that trastuzumab treatment in MBC affects both the numbers of T_reg_ cells and the frequency of circulating Th17 cells.

Most patients in this study were receiving trastuzumab monotherapy; however, a small proportion received trastuzumab in combination with docetaxel or vinorelbine. Although we did not directly address the effect of chemotherapeutic agents alone on T_reg_ and Th17 frequencies, we observed no significant differences between the patient population treated with monotherapy and those treated in combination with chemotherapy. Accordingly, Perez *et al* (2007) noted no specific difference in T_reg_ frequencies in HER2-negative breast cancer patients receiving the same chemotherapy regimen as patients receiving trastuzumab in combination, and concluded that the differences in T_reg_ frequency were attributed specifically to trastuzumab therapy. Samples were taken from participating patients throughout their treatment, and for most patients, the immune populations measured were similar throughout the period of trastuzumab therapy.

Here, we saw a reduction of T_reg_ numbers, coupled with a converse increase of Th17 cells in the PB of MBC patients receiving trastuzumab therapy. This effect was less pronounced in the adjuvant setting for reasons that remain unclear. Measurement of the frequency of immune cells, such T_reg_ and Th17 cells may prove useful in identifying whether patients are showing any positive response to treatment or not, and this would enable cessation of unnescessary therapy in unresponsive patients. This would be beneficial as trastuzumab is both expensive and can sometimes be associated with serious side effects including cardiotoxicity (Mariani *et al*, 2008). Whether direct HER2 targeting or an antibody affect is involved in the change in number of T_reg_ and Th17 cells in trastuzumab-treated HER2-positive breast cancer remains unclear. It is possible that trastuzumab may lead to the changes in the cytokine milieu or other factors that may drive expansion of Th17 cells and/or prevent the survival of T_reg_ cells in the body.

This study has been limited by small sample sizes, and functional data have not been collected. Our results, however, support the role of T_reg_ and Th17 cells in trastuzumab therapy. It would appear that further studies are required to confirm the relationship between T_reg_ frequencies and cancer as some of our data conflict with those published previously (Perez *et al*, 2007). In addition, it may be of interest to further study the effect of trastuzumab therapy in EBC with more patients. It would be of particular interest to observe whether the efficacy of adjuvant therapy in HER2-positive EBC patients could be ultimately predicted by studying the frequency and functionality of patients’ T_reg_ and Th17 cells.

These data are of interest in cancer therapy in general, as harnessing the immune system to improve responses to existing therapies is of increasing importance in clinical trial design of newer immunotherapeutics. The coadministration of trastuzumab along with therapies that either promote Th17 or reduce T_reg_ cells may be a particular direction, with the aim of ultimately improving the prognosis for patients unresponsive to trastuzumab or other therapies.
