# Supplementary figures and images for: The effects of trastuzumab on the CD4+CD25+FoxP3+ and CD4+IL17A+ T-cell axis in patients with breast cancer
Source: Br J Cancer. 2009 Mar 10;100(7):1061–7. doi: 10.1038/sj.bjc.6604963 (PMC2670001; doi:10.1038/sj.bjc.6604963)

## Slide 1
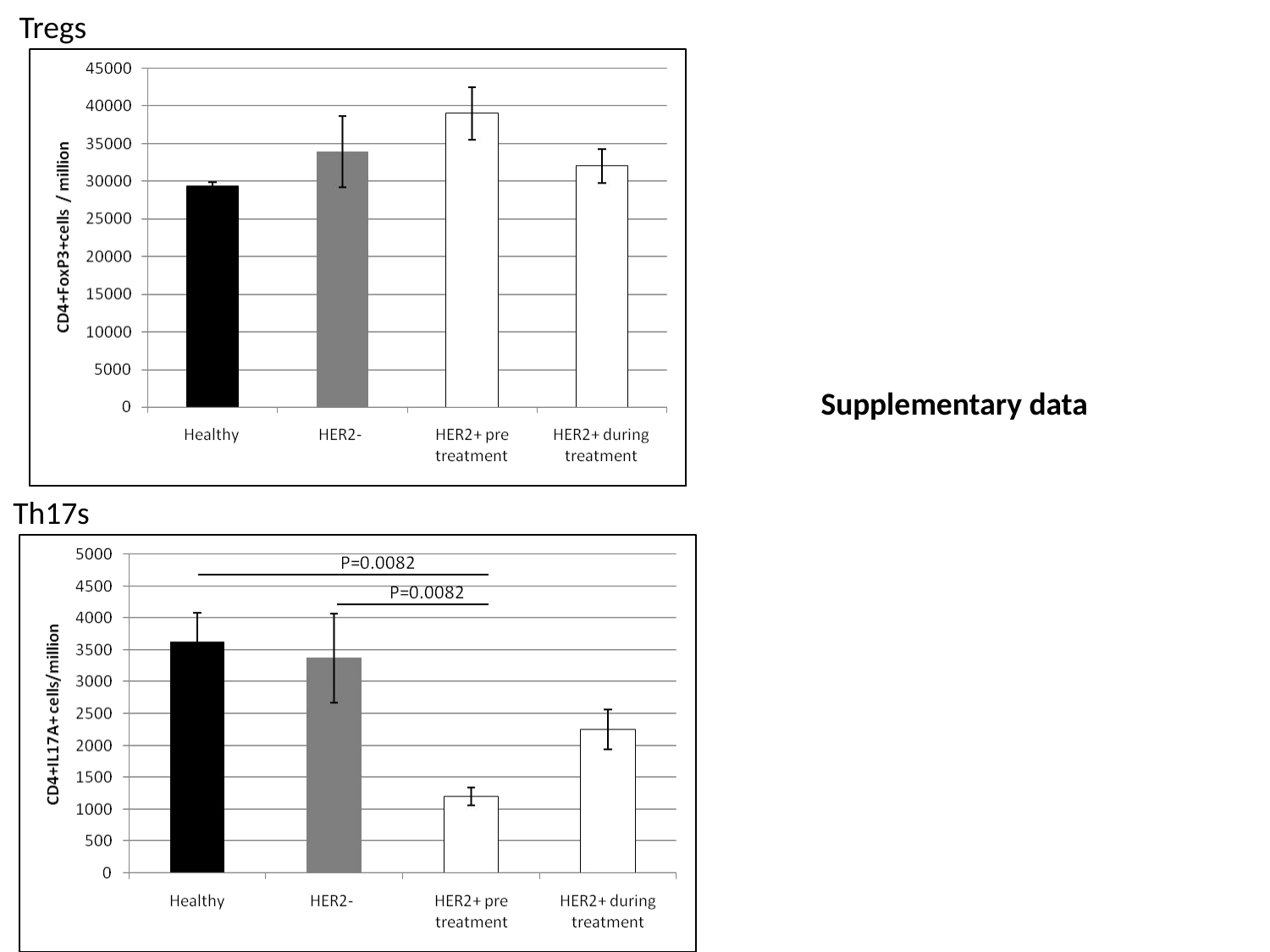

Tregs
Supplementary data
Th17s

Supplement: Supplementary figure 1 [file 6604963x1.ppt]
