# Supplementary material for: The effects of trastuzumab on the CD4+CD25+FoxP3+ and CD4+IL17A+ T-cell axis in patients with breast cancer
Source: Br J Cancer. 2009 Mar 10;100(7):1061–7. doi: 10.1038/sj.bjc.6604963 (PMC2670001; doi:10.1038/sj.bjc.6604963)
Supplement: Supplementary figure Legend [file 6604963x2.doc]

**Supplementary figure. The absolute number of Treg and Th17 cells follows the same trend as their frequency in CD4+ cells.** The absolute number of Tregs and Th17 cells was measured as the number of CD4+FoxP3+ cells (Tregs) or CD4+IL17A+ cells (Th17s) per million PBMCs analysed. Error bars represent +/- standard error. P values of less than 0.05 as measured by the Mann-Whitney U Test, were considered significant.
